# Dynamic causal modelling of anticipatory skin conductance responses

**DOI:** 10.1016/j.biopsycho.2010.06.007

**Published:** 2010-09

**Authors:** Dominik R. Bach, Jean Daunizeau, Karl J. Friston, Raymond J. Dolan

**Affiliations:** Wellcome Trust Centre for Neuroimaging, University College London, 12 Queen Square, London WC1N 3BG, United Kingdom

**Keywords:** Galvanic skin response, GSR, Electrodermal activity, EDA, Generic model, Forward model, Fear conditioning

## Abstract

Anticipatory skin conductance responses [*SCRs*] are a widely used measure of aversive conditioning in humans. Here, we describe a dynamic causal model [*DCM*] of how anticipatory, evoked, and spontaneous skin conductance changes are generated by sudomotor nerve activity. Inversion of this model, using variational Bayes, provides a means of inferring the most likely sympathetic nerve activity, given observed skin conductance responses. In two fear conditioning experiments, we demonstrate the predictive validity of the *DCM* by showing it has greater sensitivity to the effects of conditioning, relative to alternative (conventional) response estimates. Furthermore, we establish face validity by showing that trial-by-trial estimates of anticipatory sudomotor activity are better predicted by formal learning models, relative to response estimates from peak-scoring approaches. The model furnishes a potentially powerful approach to characterising *SCR* that exploits knowledge about how these signals are generated.

## Research highlights

▶ Full generative model for skin conductance changes. ▶ Inversion of this model yields estimates of sudomotor activity. ▶ In fear conditioning, estimated sudomotor activity is more closely related to the experimental manipulation than conventional *SCR* measures.

## 1 Introduction

Anticipatory skin conductance responses [*aSCRs*] are a widely used index of aversive Pavlovian conditioning (or fear conditioning) in humans (see e.g. Boucsein, 1992) much like anticipatory freezing behaviour used in animal studies. Anticipatory *SCRs* are assumed to reflect preparatory reactions to an upcoming, often aversive, event and serve as an indicator of whether conditioning was successful (e.g. in neuroimaging studies of aversive learning) (Morris and Dolan, 2004; Milad et al., 2007; Marschner et al., 2008; Delgado et al., 2008), or constitute a primary outcome measure (e.g. in studies of learning without conscious awareness) (Cornwell et al., 2007; Flykt et al., 2007). Beyond fear conditioning, *aSCRs* to upcoming rewards and punishments are important in the study of human decision making, where they may reflect characteristics of a choice situation, such as variance in expected outcomes (Tomb et al., 2002). On a trial-by-trial basis, *aSCRs* are often assumed to reflect the progression of learning, and enable empirical tests of formal learning theories (Izawa, 2008).

Thus, *aSCRs* form a methodical cornerstone of human associative learning and decision making research. Their quantification relies on detecting a peak or computing the mean response over an anticipation time window, relative to a baseline. Such approaches require a robust baseline, and therefore lengthy inter-trial intervals, a requirement not often met in cognitive neuroscience research. For example, closely spaced events in cognitive paradigms often lead to overlapping conductance responses, which are notoriously difficult to analyse (Barry et al., 1993).

Skin conductance changes are generated by sweat excretion caused by sudomotor (sympathetic) nerve activity [*SNA*]. This *SNA* has a much shorter time constant than the ensuing skin conductance responses. Thus, inferring *SNA* from observed skin conductance can, in principle, help disentangle overlapping responses. In the absence of invasive methods, SNA might be inferred using model inversion methods that map observed *SC* to underlying *SNA*. This type of inference is now commonplace in neuroimaging research, most notably as described within the framework of dynamic causal modelling [*DCM*] (Friston et al., 2003).

At the heart of *DCM* is a causal model, also referred to as a generative or forward model, which describes a mapping from underlying causes (i.e. neural states) to empirical observations (e.g. BOLD response, EEG waveform, or *SC*). In our case, this mapping SNA↦SC describes the skin conductance, given sudomotor nerve activity. Inverting this causal model yields a reverse mapping from observation to (most likely) underlying causes; in our case, the inversion SC↦SNA describes the (most likely) sudomotor nerve activity, given the model and the observed skin conductance (see Section 4). The key difference between previously proposed models for event-related skin conductance changes, where event timing is known (Lim et al., 1997; Bach et al., 2009), and the model considered here is that timing, duration, and amplitude of *SNA* bursts have to be estimated from the data. Deconvolution methods afford such estimates, as they try to recover the *SNA* time series from the skin conductance data (Alexander et al., 2005; Benedek and Kaernbach, 2009). Our approach represents an informed Bayesian deconvolution, which rests on parameterising the *SNA* in a manner that provides for a quantitative description of the underlying state. Furthermore, this allows one to model different inputs to sudomotor nerve activity, which could relate to different neural or psychological processes.

We recently proposed a *DCM* for spontaneous fluctuations in skin conductance (Bach et al., in press). Here, we generalise this *DCM* to embrace anticipatory, evoked, and spontaneous skin conductance changes. We hypothesised that explicit estimates of *SN* activity under this model would have a higher predictive validity than conventional response estimates, in the context of fear conditioning. Hence, we test the *DCM* on data from two fear conditioning experiments. To render the method widely accessible, we include it as function scr_dcm.m in the software suite *SCRalyze*, freely available under the GNU general public license from *scralyze.sourceforge.net*. This general function allows the user to specify time points for evoked responses, time windows for anticipatory, spontaneous, and baseline fluctuations; thus catering for experimental paradigms that extend the relatively simple design used here, and permitting analysis of multiple (and overlapping) anticipatory *SCRs*.

## 2 Methods

### 2.1 Forward (sudomotor) neural model

Skin conductance changes can loosely be grouped into anticipatory, evoked, and spontaneous; the latter comprising spontaneous fluctuations and slow skin conductance level [*SCL*] drifts. Physiological research has focused on evoked skin conductance responses [*eSCRs*] and spontaneous fluctuations [*SF*]. Evoked *SCRs* are generated by short sudomotor bursts that follow an event (e.g. an electric shock) with a constant latency (Nishiyama et al., 2001). On the other hand, *SF* have been investigated in somewhat greater detail and seem to be generated by *SNA* bursts of 637 ± 37 ms duration (Macefield and Wallin, 1996), although from the figures in this and other papers (Ogawa and Sugenoya, 1993; Nishiyama et al., 2001) it seems that the burst duration can extend up to 1.5–2 s.

**Fig. 1:**
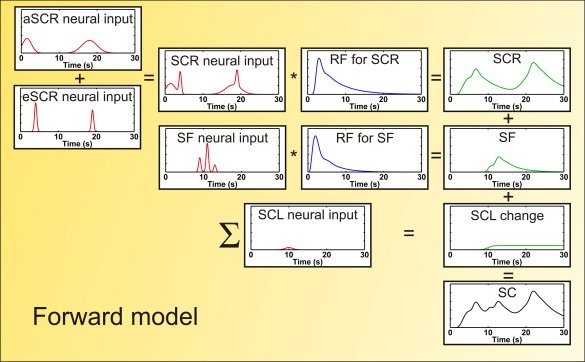
Example for how the skin conductance signal during two trials is generated (simulated data). In this example, a CS is presented at 0 and 15 s, eliciting two anticipatory sudomotor activity bursts (*aSCR neural input*). The US is received at 4 and 19 s and each time evokes a sharper firing burst (*eSCR neural input*). Both these neural input functions are summed up (*SCR neural input*) and convolved with the response function specific to the skin/sweat gland system (*RF for SCR*) to cause the observed *SCR*. Similarly, a small number of spontaneous sudomotor bursts occurs in the inter-trial interval (*SF neural input*), which is convolved with its own response function (*RF for SF*) to cause the observed *SF*. A small baseline change in the inter-trial interval is modelled here with another neural input function (*SCL neural input*) that is simply cumulatively integrated to yield the observed *SCL change*. All response components are then added to yield the observed compound skin conductance (*SC*) signal.

The present *DCM*, in line with our previous approach (Bach et al., in press), parameterises *SNA* with *neural input functions*. We define four neural input functions: *aSCR*, *eSCR*, *SF* and *SCL* drifts (see Fig. 1, plotted red) (for interpretation of the references to colour in this figure legend, the reader is referred to the web version of the article). These neural input functions reflect assumptions (prior beliefs) based on the experimental design. In particular, their specification embodies some prior knowledge about the time window during which each of these responses should occur, and what their shape is likely to be. We assume each input function is a sequence of Gaussian bump functions. Each instance of these bumps is parameterised by its amplitude, timing and duration (width). Amplitude, timing and duration are estimated trial-wise for *aSCR* within an anticipation window. Amplitude is estimated trial-wise for *eSCR*, where duration and timing (i.e. delay) are assumed to be constant across trials (and delay is estimated from a principal component analysis [PCA] of all responses each dataset). Amplitude and timing are estimated in inter-trial intervals for *SF* and *SCL*, while their duration is pre-determined, and the maximum number of *SF* is 0.5 responses/s (see Appendix A for details).

### 2.2 Forward (skin conductance) response model

No simultaneous recordings of *SC* and *SNA* have addressed how the shape of the ensuing *SF* relates to bursting, but there is evidence that the convolution kernels (impulse response functions) for *eSCRs* look slightly different than those for *SF* (Nishiyama et al., 2001; Bach et al., 2010a,b). These reports suggested that both response types (evoked and spontaneous) can be modelled as product of a linear time-invariant system (i.e. with time-invariant kernels or response functions). They also describe the implicit impulse response functions that reflect the canonical shape of observed *eSCR*/*SF* at a phenomenological level (i.e. not derived from a biophysical model, but from physiological observations).

In light of these observations, our *DCM* models the mapping from *SNA* to *SC* as a linear time-invariant convolution, which is described completely by its *impulse response function* [*RF*]. Since physiological evidence suggests that these response functions are different for *eSCR* and *SF*, they are modelled by separate *RFs* (see Fig. 1, plotted blue), while we assume the same *RF* for *eSCR* and *aSCR* (i.e. their neural input components are added before convolution with the *RF*, see *SCR neural input*, plotted red in Fig. 1). Both *RFs* are assumed to be constant across trials. The *RF* for *SF* is determined using *a priori* forms from the literature (Bach et al., 2010b), while the *RF* for *eSCR*/*aSCR* is determined using a PCA of all responses in each dataset. Finally, we model *SCL* drifts that survive high-pass filtering (possibly caused by peripheral factors and filter artefacts of no interest) with a *RF* that simply accumulates (integrates) the value of the corresponding neural input function. The *SCR* components, plotted green in Fig. 1, are then added up to form the skin conductance time series, plotted black in Fig. 1.

### 2.3 Model inversion

**Fig. 2:**
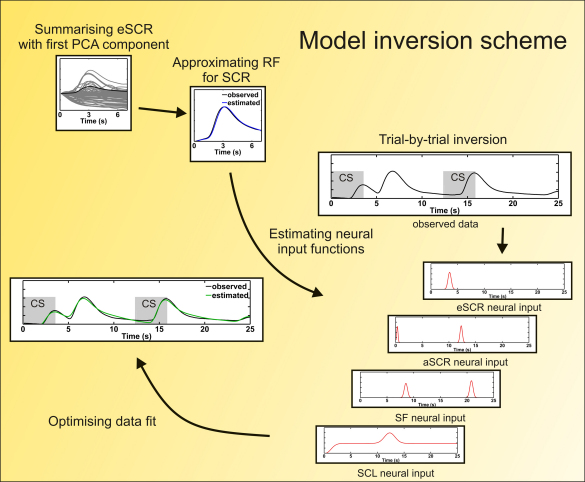
Schematic summary of the model inversion scheme. First, all *eSCRs* from one dataset are summarised by their first principal component, and a response function (i.e. the *RF for SCR*) is approximated to this data. This, together with the *RF for SF*, knowledge about the timing of experimental events, and assumptions about the form of the neural input, is then used to estimate the most likely underlying neural inputs, given the data. These neural input functions are estimated to optimise the data fit.

Model inversion is described in Appendix A in detail. In summary, Fig. 2 shows that for each participant, responses to the US and omission of US were summarised by their first principal component. Parameters for the *RF* for *eSCR*/*aSCR* were estimated to approximate this average response. On a trial-by-trial basis, the model was then inverted to estimate the different neural inputs, given the observed data and the *RFs*.

### 2.4 Design and participants

We used classical (Pavlovian) learning in a discriminant delay conditioning task. In two experiments, participants learned contingencies between a conditioned stimulus [CS+] that co-terminated with an aversive unconditioned stimulus [US] 50% of the time, and a second CS− that predicted the omission of the US. In the first of two experiments, stimulus onset asynchrony [SOA] between CS and US was an additional between subjects factor with three levels, while it was held constant in the second experiment. US type, incidental task, inter-trial interval [ITI], and trial number, were different between both experiments. We recruited healthy unmedicated participants from the general population who received monetary compensation for their participation. 32 individuals (16 male, 16 female, mean age ± standard deviation: 22.4 ± 4.6 years, range 18–34 years) took part in experiment 1, and an independent sample of 20 individuals (10 male, 10 female, mean age ± standard deviation: 22.2 ± 4.0 years, range 18–30 years) participated in experiment 2. All participants gave written informed consent, and the study was approved by the local ethics committee.

### 2.5 Stimuli and apparatus

#### 2.5.1 Experiment 1

CSs were a blue and an orange filled circle that could appear on each trial on the left or on the right of screen centre. Participants were asked to indicate the position of the circle with the left and right cursor buttons. One of the two colours (balanced across participants), predicted a US with a contingency of 50%. The US was a 1 s burst of white noise (10 ms onset and offset ramp, ∼95 dB sound pressure level), delivered via headphones (PX-660 Pro Luxe, Fujikon, Hong-Kong, China). SOA between the CS und US was varied between participants to be 4, 10, or 16 s. The ITI was selected randomly on each trial from 14, 19, or 23 s. There were 64 trials, 32 for each CS type with the whole experiment lasting between 30 and 45 min (depending on the CS/US-SOA).

#### 2.5.2 Experiment 2

The same CS as above appeared in the centre of the screen. Participants were asked to indicate the colour with the cursor up/cursor down key. Colour-key and colour-CS associations were balanced across participants. The US was an uncomfortable electric shock, delivered via a pin-cathode/ring-anode configuration attached to the dominant forearm. The shock was a 500 Hz current train with individual square pulse width of 0.5 ms, varying current amplitudes (mean ± SD: 0.90 ± 0.63 mA) for 500 ms. Before the experiment, discomfort and pain thresholds were assessed with increasing stimulation intensity and stimulation intensity was set just below the pain threshold. The SOA between CS and US was 3.5 s. ITI was randomly determined on each trial to be 7, 9, or 11 s. At the end of a few randomly selected trials (10 CS−, 5 CS+ with US, 5 CS+ without US), participants were asked to rate “’How likely did you think you would get a shock?” using a horizontal visual analogue scale [VAS] from 0% to 100%. There were 180 trials, 90 for each CS type with the whole experiment lasting about 45 min.

#### 2.5.3 Common settings

After each experiment, participants were shown the CS one at a time and asked “how likely is it that a loud tone” (experiment 1) or “how likely is it that a shock” (experiment 2) “would be delivered after that symbol” on a horizontal VAS from 0% to 100%. Then, they were shown both CS at the same time and asked which one they “liked better”. Both experiments were programmed in Cogent (Version 2000v1.25; www.vislab.ucl.ac.uk/Cogent) on Matlab 6.5 (MathWorks; Natick, MA; USA).

Skin conductance was recorded as described previously (Bach et al., 2009, 2010a) on thenar/hypothenar of the non-dominant hand using 8 mm Ag/AgCl cup electrodes (EL258, Biopac Systems Inc., Goleta, CA, USA) and 0.5%-NaCl electrode paste (GEL101; Biopac). Constant voltage (2.5 V) was provided by a custom-build coupler, whose output was converted to an optical pulse with a minimum frequency of 100 Hz at 0 μS to avoid aliasing, digitally converted (Micro1401, CED, Cambridge, UK), and recorded (Spike2, CED). Temperature and relative humidity of the experimental room was between 18–25 °C and 31–51% for both experiments.

### 2.6 *SCR* analysis

Data analysis was implemented in Matlab using custom code available from the authors. Prior to analysis, skin conductance data were converted back to a waveform signal with 100 Hz time resolution, filtered with a bidirectional first order Butterworth band pass filter with cut-off frequencies of 5, and 0.0159 Hz (corresponding to a time constant of 10 s), respectively, and down-sampled to 10 Hz sampling rate. The entire *SCR* time series was then *z*-transformed to account for inter-individual differences in responsiveness which might be due to peripheral factors alone (see Bach et al., 2009). Anticipatory reactions were modelled as single responses (entire interval response, EIR). For lengthy SOAs it is common practice to analyse the first and second half of the anticipation window separately (first and second-interval response, FIR/SIR), although there is little theoretical justification for this and recent work challenges its validity (Pineles et al., 2009). We therefore modelled one response for each half of the longer SOAs (10 and 16 s) and asked empirically whether this provided a better model of the data.

We benchmarked our method against two other analyses. First, peak measures for each trial were extracted as the maximal *SCR* value during the full anticipation period (entire interval response, EIR), or for both halves of the anticipation period (first and second-interval response, FIR/SIR) separately, all corrected for a baseline period of 1 s before CS presentation. For SOAs under 5 s, we extended the peak window until 5 s after CS onset to account for *SCR* latency.

As a second benchmark, we used a general linear convolution model [GLM] analysis (Bach et al., 2009) under an assumption that the neural functions were very short compared to the *SCR* response functions. Each event onset (or onset of each half of the anticipation window, respectively) was modelled as a stick function, convolved with a canonical response function (Bach et al., 2010a). There were either 4 event types (CS−, CS+ not followed by US, CS+ followed by US, US), or one event type per CS and US per trial to allow trial-by-trial deconvolution. In an additional analysis we modelled the canonical response function and its derivatives and recovered the peak of the estimated response (as previously proposed for fMRI analysis) (Calhoun et al., 2004; Worsley and Taylor, 2006). This latter approach provides maximum flexibility for modelling individual responses in the GLM framework and is yet subtly different from *DCM*: our *DCM* uses the same conductance response function for all trials but allows for trial-specific variations in the underlying neural input. Conversely, for trial-specific GLMs using a set of basis functions, the response function can vary from trial to trial. This is because the GLM effectively composes neural and conductance response functions together and is unable to disambiguate between differences in neural input and differences due to differences in the response function (see Lim et al., 1997 for a similar approach).

### 2.7 Statistical inference

For analysis of CS effects, we averaged, for each participant, estimated responses to CS− on the one hand and those to the CS+ that were not followed by a US on the other hand. These response estimates were the inferred peak responses associated with each trial type. For the *DCM* analysis these peak amplitudes were of inferred sudomotor activity, for the alternative methods these reflect the *SCR* peak amplitude. These measures were used as subject-specific summaries and analysed with a 2 (CS) × 3 (SOA) ANOVA (experiment 1), and with a one-way ANOVA (experiment 2).

This analysis only allows an inference about whether each response measure is significantly related to the experimental manipulation (CS+/CS−); however, we also asked if any response measure had a stronger association with the experimental manipulation than other measures. We framed this question in terms of model comparison by quantifying how well different response measures predict, for each participant, the conditions (CS+ or CS−) the responses were elicited under. To do this, we used general linear models (GLMs), where the contrast vector for CS+/CS− was the predicted variable and the predictors were a response measure and subject-specific terms (accounting for mean response differences between subjects, independent of the contrast CS+/CS−). Inverting these GLMs yields a squared error, which will be smaller when a response measure better predicts the experimental conditions. To formalise this, we used Bayes factors that quantify how much more evidence there is for a GLM with one response measure relative to another. Under the assumption that the errors are normally distributed, the following equality holds:

$$BIC=\text{log}(\sigma_{e}^{2})+\frac{k}{n}\text{log}(n)$$

where *BIC* is the Bayesian information criterion, $\sigma_{e}^{2}$ is the error variance of the GLM, *k* the number of predictor variables, and *n* the number of data points. The Bayes factor (*BF*) for each pair wise model comparison is then given by

$$\text{log}(BF)=BIC_{\text{mod}1}-BIC_{\text{mod}2}=\text{log}(\sigma_{e(\text{mod}1)}^{2})-\text{log}(\sigma_{e(\text{mod}2)}^{2})$$

In the tables, we state log-Bayes factors, which quantify the evidence for one model relative to another (*DCM*). In this context, a log-Bayes factor of 3 indicates that one response measure is $e^{3}≅20$ better (more likely), when predicting the experimental condition.

To determine whether *aSCR* measures relate to the underlying learning processes, we tested whether their evolution could be explained by a formal learning model. We used a simple Rescorla–Wagner learning algorithm (Rescorla and Wagner, 1972): $V_{CS}(t)=V_{CS}(t-1)+\alpha_{CS}[\lambda (t)-V_{CS}(t-1)]$, where *V*_CS_ is the associative strength for the CS− or CS+ on the *t*-th trial, *α* is a CS− specific learning rate, and *λ* denotes whether or not the US was realised on any given trial (0 or 1). We used initial values of 0.5 for each CS, and estimated *α* from each participant's data, using an ordinary least square criterion and gradient search. *V* was assumed to be linearly related to *aSCR*. We then set up regression models for each subject, where we tried to predict the response measure using the learning model, under optimal parameter values. The explained variance with each of these models, *R*^2^, was then averaged over subjects, for each response measure. This served to illustrate the face validity of the *DCM* estimates. Because the response measure (predicated variable) changed between the measures, we did not pursue a direct comparison of these regression models. Implementation of a Pearce–Hall learning rule (Pearce and Hall, 1980) yielded similar results, in terms of explained variance in the alternative *aSCR* measures.

## 3 Results

### 3.1 Awareness of the conditioning procedure

At the end of experiment 1, participants rated the US probability after the CS+ higher than after the CS− (mean ± standard error: 56.3% ± 3.6% vs. 12.2% ± 4.3%; *t*_31_ = 7.3; *p* < 0.0001). When asked which CS they preferred, 21 of 32 participants preferred the CS− over the CS+ (binomial test: *p* < 0.05). Similar results were found in the second experiment (expectancy: 75.8% ± 2.0% vs. 5.9% ± 2.8%; *t*_19_ = 17.6; *p* < 0.0001; preference: 17/20, *p* = 0.001). Expectancy ratings during the course of experiment 2 also revealed a main effect CS+ > CS− (68.8% ± 3.7% vs. 32.1% ± 4.0%; *t*_19_ = 5.9; *p* < 0.0001) and a time-decreasing shock expectancy for both CS+ and CS− (main effect time: *t*_19_ = −2.7; *p* < 0.05; interaction CS × time: *t*_19_ = 0.8; *p* > 0.40). There was no effect of CS on reaction times.

### 3.2 Summarising response estimates

Sudomotor nerve parameters from the model inversion are summarised in Table 1. Across CS− and CS+, estimated response amplitudes are in a plausible range, and responses to the US are higher than responses to the CS. Estimated response latencies to the US are consistent with those reported previously (Bach et al., 2010a), and the number of SF in the inter-trial interval are equally in a plausible range (Bach et al., in press). The parameter values thus provide evidence for the physiological plausibility of the model. Note that all inferences were based on *z*-transformed data (Table 2).

The predictive validity of the different anticipatory measures was assessed by their ability to detect difference elicited by our experimental manipulation involving a contrast of CS+ versus CS−. There was no consistent advantage from modelling first and second-interval response separately, i.e. the sensitivity to differentiate between CS− and CS+ was not consistently higher for any response measure when modelling two responses per anticipation window, such that we report only results for the entire interval response (note that this only addresses the sensitivity to detect CS+/CS− differences, not the true underlying sudomotor activity, see Section 4).

Table 2 shows that only *DCM* estimators predicted a main effect of CS for experiment 1, while all other measures failed to detect this effect. In experiment 2, all measures detected the effect of CS, with *DCM* showing the greatest sensitivity. Consequently, for both experiments, *DCM* had a significantly higher predictive validity than any of the other measures, as approximated by Bayes factors. These Bayes factors express how much more evidence there is for one model as opposed to another one, as approximated from the residual error. Models here correspond to predicting CS from *SCR* measures. Given that the smallest log-Bayes factor encountered is 56, there is at least $e^{56}≅2\times 10^{24}$ times more evidence for the statement that *DCM* parameters predict CS than for any other measure. Note that in experiment 1, where CS/US-SOA was varied between 4 and 16 s, there was no influence of SOA on the sensitivity of the *DCM* estimates (or on response estimates from any of the other methods), i.e. no CS × SOA interaction.

**Fig. 3:**
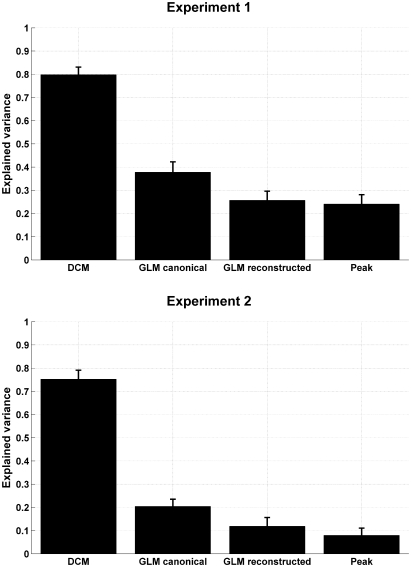
Variance ratio *R*^2^ of the *aSCR* estimates that can be explained by formal learning models (i.e. Rescorla–Wagner) under optimal parameters, suggesting that *DCM* estimates bear a closer relation to central processes than estimates from GLM with one regressor per trial, or peak-scoring estimates.

While having higher predictive validity than alternative measures, the real power of our *DCM* lies in trial-by-trial estimates of response amplitudes, which is not captured by a contrast between the two conditions. To assess the face validity of the alternative peak summaries, we assumed that learning could be described by formal learning theory. Using a simple Rescorla–Wagner learning rule, with two learning rates (for CS+ and CS−), we fitted each participant's dataset and computed the explained variance under an optimal learning rate (see Section 2 for details). For the different measures, Fig. 3 shows the ratio of explained variance *R*^2^, and provides evidence that between-trial *DCM* estimates were consistently explained by the Rescorla–Wagner rule, while only small fractions of the between-trial variance in the alternative estimates were explained. This suggests that our *DCM* estimates comply with predictions from formal learning theory, which captures a host of animal behaviours (Rescorla and Wagner, 1972) and evoked neural responses in humans during learning (see e.g. Ploghaus et al., 2000; Glascher and Buchel, 2005; den Ouden et al., 2009).

## 4 Discussion

We have described a dynamic causal model for *SC* changes that includes anticipatory, evoked, and spontaneous skin conductance changes and allows, via model inversion, estimation of the most likely neural contributions to each of these components. For estimates of *aSCR*, we show that this approach has higher predictive validity than conventional (peak scoring) analysis, or the previously proposed GLM (Bach et al., 2009). The latter proved successful for analysing *eSCRs* (for an application see e.g. Talmi et al., 2009) but does not model variable delay and duration of *aSCRs* with sufficient constraints. In particular, we show that the *aSCR* amplitude, as estimated by *DCM* inversion, discloses CS+ and CS− effects more sensitively than measures derived from other more conventional methods.

The validity of our approach is reinforced by the observation that, on a trial-by-trial basis, these estimates are closely related to predictions from a formal learning model that provides a good account of animal learning behaviour and evoked neural activity measured in humans during learning (see e.g. Ploghaus et al., 2000; Glascher and Buchel, 2005; den Ouden et al., 2009). Thus, it appears that *DCM* provides a good trial-by-trial quantification of sympathetic activity and links peripheral psychophysiology to the underlying generative neural processes.

An important factor which may account for the greater predictive validity of our method is a robustness to random fluctuations that is conferred by formal model constraints (i.e. parameterisation of the unknown *SNA*). This contrasts with previous deconvolution approaches that try to recover unconstrained *SNA* estimates (Alexander et al., 2005; Benedek and Kaernbach, 2009) although it needs to be explored whether there are circumstances under which the strong model constraints impede accurate inference. A potential limitation of the present *DCM* is that it requires filtering of data such that they comply with model assumptions about the filtered data-features. Recent deconvolution approaches have attempted to make inference from the unfiltered *SC* time series (Benedek and Kaernbach, 2009). Our model only accounts for residual baseline changes and filter artefacts, and we suspect that it could be further improved by more precisely modelling tonic skin conductance, which would make filtering unnecessary. More generally, although we have tried to substantiate the physiological plausibility of the *DCM* presented here by examining a wide range of model parameters, we suspect its accuracy can be improved by more informed physiological knowledge. Among other caveats, no complete biophysical model of skin conductance generation is available at present, and in particular it is unknown whether apparent differences between evoked and spontaneous skin conductance changes are due to different neural input or different response functions. Indeed, one of the advantages of dynamic causal modelling is that one can evaluate a new model in relation to an old model using model evidence (i.e. Bayesian model comparison). This provides a principled way to evaluate changes in the form of the model or changes in its priors that encode physiological constraints. This may be particularly important if one has access to parallel neurophysiological data that place informed constraints on the mapping from sudomotor nerve activity to skin conductance responses.

The particular *DCM* presented here offers considerable potential for generalisation. First, many analyses of *aSCR* assume multiple sudomotor bursts in the anticipation window. For simplicity, we model multiple bursts with one Gaussian bump function that can have more, or less, dispersion. We also modelled two responses for longer anticipation windows and did not find an improvement in predictive validity. This simply implies that our model is sufficiently accurate to infer differences in sympathetic activity but this does not speak to the form of the true underlying physiology. While we approximate sudomotor firing in the anticipation window with a Gaussian bump of several seconds duration, the true firing pattern is probably a train of repeated bursts. The benchmark for testing the underlying physiology would be the model likelihood (i.e. evidence), given the data. Our relatively small dataset (10 participants for each of the longer anticipation windows) does not provide sufficient sensitivity for such model comparisons. While *DCM* formulation allows such analyses, more (precise) data would be needed to disambiguate between alternative models of anticipatory bursting. Similarly, our *DCM* can be applied to more complex experimental designs in decision making where multiple events occur in quick succession, or to *eSCR* paradigms with long event duration where the exact time point of the response is unknown.

Separating overlapping *SCRs* in long ITI experiments has been a major motivation for model-based analysis (Barry et al., 1993). There are however more fundamental reasons why model-based analysis is useful. In psychophysiology, formal statistical inference is often performed on observable quantities (e.g. skin conductance), and from such results, unobservable quantities (i.e. psychological processes) are inferred. This approach is only meaningful if a conclusion is built on a model of how observable and unobservable quantities relate to each other. Such models are usually implicit in the way conclusions are drawn. We have shown in several ways that it is possible to mathematically explicate and test such models (Bach et al., 2009, in press, 2010a,b). Having established such models we are now in a position to make statistical inference on unobservable quantities of interest, for example anticipatory neural activity in the case considered above.

Non-linear models allow considerable flexibility for capturing dynamic biophysical relations. Dynamic causal modelling (Friston et al., 2003; Daunizeau et al., 2009a) is now standard in neuroimaging, with widespread applications in the analysis of fMRI, EEG/MEG (David et al., 2006; Chen et al., 2008; Kiebel et al., 2009; Penny et al., 2009; Daunizeau et al., 2009b), and electrophysiological data (Moran et al., 2009). The power of such approaches lies in a precise formulation of the mapping from underlying causes to empirical observations. This mapping enables one to place biophysical constraints on the models and its associated estimators. Furthermore, the parameters and states of these models have a direct and useful biological interpretation.

In the context of neuroimaging, *DCM* is most often used to infer causal interactions between regions of neural activity, and to estimate connection strengths between these nodes. Here, we present a novel application of *DCM*, where the causal structure between two nodes is assumed to be known (i.e. a neural input influences skin conductance), but where neural contributions are temporally separable. Thus, this *DCM* allows trial-by-trial estimates of different neural contributions to one observed variable.

More specifically, we now have a model that describes how different neural inputs map on to skin conductance responses, which affords estimates of various components of *SNA*, given observed *SC* data. We show that this model is efficient when analysing anticipatory *aSCRs* in the context of aversive conditioning, and has higher predictive and face validity than previously proposed characterisations.

## Figures and Tables

**Table 1:** Estimated parameters of the sudomotor nerve activity across participants for the two experiments. All amplitudes are expressed in sudomotor units, where one unit is defined as the sudomotor burst amplitude that leads to an *eSCR*, *SF*, or *SCL* change of 1 μs peak amplitude. Conditioned and unconditioned reactions (CR and UR) are both expressed in *eSCR* units, but the ensuing conditioned skin conductance response also depends on the dispersion of the sudomotor burst which is variable for CR and reported separately.

	Experiment 1	Experiment 2
	Mean ± SEM	Mean ± SEM
CR− amplitude (units)	0.37 ± 0.08	0.26 ± 0.05
CR+ amplitude (units)	0.42 ± 0.09	0.33 ± 0.09
*z*-Transformed CR− amplitude (*z*-units)	1.19 ± 0.12	1.02 ± 0.06
*z*-Transformed CR+ amplitude (*z*-units)	1.30 ± 0.15	1.24 ± 0.09
CR− dispersion (SD in s)	2.14 ± 0.25	1.22 ± 0.07
CR+ dispersion (SD in s)	2.01 ± 0.26	1.06 ± 0.06
CR− peak latency (s)	2.56 ± 0.56	0.51 ± 0.06
CR+ peak latency (s)	2.52 ± 0.66	0.88 ± 0.11

UR amplitude (units)	1.89 ± 0.35	1.87 ± 0.32
*z*-Transformed UR amplitude (*z*-units)	8.07 ± 1.34	10.72 ± 1.68
UR latency (s)	1.95 ± 0.14	1.75 ± 0.11

SF frequency (SF > 0.1 units, in Hz)	0.067 ± 0.005	0.135 ± 0.008
SF amplitude (SF > 0.1 units, in units)	0.29 ± 0.01	0.29 ± 0.01

SCL change frequency (SCL change > 0.1 units, in changes/trial)	0.11 ± 0.01	0.14 ± 0.01
SCL change amplitude (SCL change > 0.1 units, in units)	0.17 ± 0.01	0.19 ± 0.01

**Table 2:** Main effect of CS from individual repeated-measures ANOVAs for each alternative estimate, and comparison with *DCM* estimates. *DCM* reliably detects the CS+ > CS− effect and makes a significantly better prediction than other estimates. Rows: Scoring of *aSCRs*: *DCM* – estimates from inversion of a dynamic causal model. Peak – peak within the anticipation window minus baseline value. GLM (conditions) – general linear model with one regressor per condition. GLM (trials: canonical) – general linear model with one regressor per trial to accommodate between-trial variance. GLM (trials: reconstructed) – general linear model with derivatives of the canonical response function, reconstructing the estimated response per trial to suppress latency-induced amplitude bias. Columns: The first pair of columns reports the conventional ANOVA and *p*-values for a test of the main effects of CS. The third column shows the logarithmised Bayes factor which indicates the logarithm of how much more evidence there is for the statement that *DCM* estimates predict CS than for the alternative *SCR* estimates.

	Experiment 1			Experiment 2		
	CS+ > CS−		Comparison with *DCM*	CS+ > CS−		Comparison with *DCM*
	*F*_1,29_	*p*	Log-Bayes factor	*F*_1,18_	*p*	Log-Bayes factor
*DCM*	5.4	<0.05		15.5	<0.001	
Peak	<1	n.s.	64	5.7	<0.05	823
GLM (conditions)	<1	n.s.	94	9.2	<0.01	56
GLM (trials: canonical)	<1	n.s.	89	7.8	=0.01	148
GLM (trials: reconstructed)	<1	n.s.	99	4.3	=0.05	2554

## Appendix A

Our generative model involves two functions that are composed to provide a complete mapping between neuronal inputs from the central nervous systems to observed skin conductance responses:

(1) *Neural input functions:* Each of *i* = 1, …, *n* SNA bursts pertaining to *j* = 1, …, 4 sudomotor components are modelled with a Gaussian bump function with amplitude $a_{i}^{\left(j\right)}\in \theta^{\left(j\right)}$, standard deviation $\sigma_{i}^{\left(j\right)}\in \theta^{\left(j\right)}$, and time of maximum firing $\tau_{i}^{\left(j\right)}\in \theta^{\left(j\right)}$. These correspond to evoked, anticipatory, spontaneous, and drift components. The input function also accounts for the physiological delay between sudomotor firing and skin conductance change with the parameter $\tau_{0}^{\left(j\right)}$. The sum of Gaussian bursts over time forms the parameterised neuronal input for each component:
  $$u^{\left(j\right)}:j=1,\ldots ,4:$$
  $$u^{\left(j\right)}(t)=\sum_{i=1}^{n}a_{i}^{\left(j\right)}\text{exp}\left(-\frac{{\left(t-\tau_{i}^{\left(j\right)}-\tau_{0}^{\left(j\right)}\right)}^{2}}{2\sigma_{i}^{(j)2}}\right)$$
  The parameters $\theta^{\left(j\right)}$ of this input function were either fixed using full or empirical priors, or estimated from the data on a trial-by-trial basis.
(2) *Response functions:* For *aSCR*/*eSCR* and *SF*, the skin conductance time series *x* is thought to result from a convolution operation applied to the sudomotor nerve activity. This can be modelled as a third-order ordinary differential equation [ODE] with fixed parameters $\vartheta^{\left(j\right)}⊃\vartheta_{i}^{\left(j\right)}:i=1,2,3$; $\vartheta^{\left(1\right)}=\vartheta^{\left(2\right)}$ where *j* refers to the different neuronal response components:
  $${\dddot{x}}^{\left(j\right)}+\vartheta_{1}^{\left(j\right)}{\ddot{x}}^{\left(j\right)}+\vartheta_{2}^{\left(j\right)}{\dot{x}}^{\left(j\right)}+\vartheta_{3}^{\left(j\right)}x^{\left(j\right)}-u^{\left(j\right)}=0:j=1,2,3$$
  The solution to the ODE was obtained from a generic numerical integrator. The parameters of the response function were fixed (either using values from the literature or estimating them from the combined data from all trials) and assumed to be constant across trials. To model baseline drifts, we assumed ${\dot{x}}^{\left(4\right)}=u^{\left(4\right)}$. Finally, the four hidden sudomotor states generate observed *SC* time series *y* with the following (trivial) observation function:
  $$y=x^{\left(1\right)}+x^{\left(2\right)}+x^{\left(3\right)}+x^{\left(4\right)}+\varepsilon$$
  where *ɛ* is a residual error term.

The fixed form and parameters of this *DCM* were determined as follows:

(a) *Event-related SCR (eSCR and aSCR):*
  • In each of *n* trials, evoked sudomotor firing was assumed to follow each of *n* unconditioned stimuli/omission of unconditioned stimuli with pre-determined delay $\tau_{i}^{\left(1\right)}=0:i=1,\ldots ,n$ and dispersion $\sigma_{i}^{\left(1\right)}=0.3s:i=1,\ldots ,n$ such that only the amplitude was estimated from the data:
    $$\theta^{\left(1\right)}⊇a_{i}^{\left(1\right)}>0:i=1,\ldots ,n$$
  • Each of *n* anticipatory sudomotor bursts, following a cue presentation at *t*_*i*_ was assumed to be variable in delay and dispersion, such that all three parameters were optimised. The timing of maximal firing was within the anticipation window, and the standard deviation could not extend SOA/2 (and analogously for the first and second half of the anticipation window when these were modelled separately):
    $$\theta^{\left(2\right)}⊇\left\{a_{i}^{\left(2\right)}>0,t_{i}<\tau_{i}^{\left(2\right)}<(t_{i}+\text{SOA}),0.1<\sigma_{i}^{\left(2\right)}<\frac{\text{SOA}}{2}\right\}:i=1,\ldots ,n$$
    Both components were assumed to obey the same response function ($\vartheta^{\left(1\right)}=\vartheta^{\left(2\right)}$), the parameters of this response function and the parameter $\tau_{0}^{\left(1\right)}=\tau_{0}^{\left(2\right)}$ that accounts for the physiological delay between sudomotor firing and ensuing *SCR* were estimated from the pooled data across all trials according to the following scheme:
  • Estimate $\tau_{0}^{\left(j\right)}$ and $\vartheta^{\left(j\right)}:j=1,2$ from the first principal component of all evoked responses (i.e. to any event, including US as well as the omission of the US), using the variational Bayes scheme described below.
  • Use these values as starting points for a second estimation where the compound response (*aSCR* and *eSCR*) is estimated from the first principal component of all event-related responses.
(b) *Spontaneous fluctuations* (*SF*)*:* For each ITI, *SFs* were modelled as proposed previously (Bach et al., in press) every 2 s such that the timing of each response was constrained to be at least 5 s after the last event and 2 s before the next cue presentation. Pre-determining delay at $\tau_{0}^{\left(3\right)}=0$ and dispersion at $\sigma_{i}^{\left(3\right)}=0.3s:\forall i$, amplitude and timing were estimated from the data:
  $$\theta^{\left(3\right)}⊇a_{i}^{\left(3\right)}>0:i=1,\ldots ,n$$
  For the response function, we used parameters from a canonical spontaneous response function as derived previously (Bach et al., in press):
  $$\left\{\begin{array}{c} \vartheta_{1}^{\left(3\right)}=2.1594\\ \vartheta_{2}^{\left(3\right)}=3.9210\\ \vartheta_{3}^{\left(3\right)}=0.9236 \end{array}\right)$$
(c) *Residual baseline changes:* One baseline change was allowed for each of *n* ITIs. All parameters for the input function were estimated from the data, and the delay parameter was set to $\tau_{0}^{\left(4\right)}=0$:
  $$\theta^{\left(4\right)}⊇\{a_{i}^{\left(4\right)},\tau_{i}^{\left(4\right)},\sigma_{i}^{\left(4\right)}\}:i=1,\ldots ,n$$

### A.1 Model inversion

The *DCMs* were inverted using a variational Bayesian inversion scheme described in Friston et al. (2007). Because processing limits do not allow inversion of the whole time series, we proceeded trial-wise in an iterative scheme. Specifically, two trials were estimated at the same time to accommodate the impact that responses in the second trial could have on estimation of parameters for the first trial. Parameters for the first trial were then extracted, while those from the second trial were used as starting values for the next iteration. At each iteration, model inversion entailed:

• Under Gaussian assumptions about the residual errors in the observation process, defining a likelihood function p(y|θ,ϑ), which measures the likelihood of a set of observations *y*, given the parameters.
• Defining *priors* p(θ,ϑ|m) on the model parameters which enable one to derive the *posterior* probability density function over the evolution parameters:
  (3) p(θ,ϑ|y)∝p(y|θ,ϑ)p(θ,ϑ).
  The posterior p(θ,ϑ|y) measures how likely any particular value of the unknown parameter is, given the measured *SC* times-series.
• Having estimated the unknown parameters of the model, we can then define an estimator $\hat{u}$ of the unknown time series of sudomotor nerve activity:
  (4) $$\hat{u}=E[u(\theta ,\vartheta )|y]$$
  where the expectation is taken under the posterior above.
